# Design, Analysis, and Verification of an Electro- Hydrostatic Actuator for Distributed Actuation System

**DOI:** 10.3390/s20030634

**Published:** 2020-01-23

**Authors:** Yang Li, Zongxia Jiao, Zimeng Wang

**Affiliations:** 1 National Key laboratory on Aircraft Fight Control, School of Automation Science and Electrical Engineering, Beihang University, Beijing 100191, China; zxjiao@buaa.edu.cn (Z.J.); buaawzm@126.com (Z.W.); 2 Ningbo institute of Technology, Beihang University, Ningbo 315800, China

**Keywords:** distributed actuation, direct drive, linear pump, collaborative rectification, modulation control

## Abstract

In order to provide a simplified and low-cost solution of the terminal for a distributed actuation system, this paper proposes an electro-hydrostatic actuator (EHA) based on the linear drive principle. The proposed actuator is directly driven by a linear pump with a collaborative rectification mechanism, whose performance relies on the collaboration of the internal two units. A pair of linear oscillating motors are employed to drive the two pump units respectively. The control of the actuator is based on the modulation of the oscillating amplitude, frequency, and phase difference of the two motors. The advantage of this actuator is that no more valve control is needed to rectify the linear pump besides the high efficiency of the direct pump drive. In this paper, both schematic and detailed structure of the actuator is presented. The kinematic and dynamic characteristics are analyzed and modeled, based on which the control method is proposed. The experiments verify the validity of the actuator structure and control.

## 1. Introduction

As an important part of the advanced flight actuation system, electro-hydrostatic actuators (EHAs) have been widely researched, developed, and successfully used on several models of commercial and military aircrafts [1,2,3]. EHAs implement the idea of distributed actuation. A heavy central power station and complex transmission pipelines are replaced by a distributed lightweight power module and networked electrical wiring, which simplifies the system architecture and improves reliability [4,5,6]. With the development of EHAs, the application scenario has expanded from aerospace into other industries such as robot, artificial limb, etc. [7,8,9]. There have been explorations and innovations in EHA design and composition. Kargov et al. employed a gear pump to compose the actuation system for artificial hands [10]. For industrial robots, Alfayad et al. proposed a neatly designed hydrostatic actuator to achieve smooth actuation with high servo stiffness [11]. Furthermore, various materials and configurations are employed in EHAs for emerging fields. Cacucciolo et al. proposed a stretchable pump based on the electrohydrodynamic principle. The pump drives a soft actuator to actuate fluidic muscle [12]. Li et al. developed a biohybrid pump using polydimethylsiloxane (PDMS). The pump generates unidirectional flow powered by engineered skeletal muscle tissue and can be used in many biological applications [13].

The pump in an EHA is the key energy transformation component. Due to its much simpler structure and lower manufacturing costs, linear pumps are always attractive options for an EHA integrator. Thanks to the development of linear drive technology, researchers can explore many related solutions in designing an EHA [14]. Linear pumps using smart materials and electromagnets can achieve very high reciprocating motion frequency and cooperate with active or passive rectification valves to provide fluid power to the actuation system [15,16,17]. The requirements of bidirectional drive and high rectification efficiency make the active valves preferred alternatives [18,19]. The valves need to be controlled in coordination with the pumping cylinder based on the sensing of the cylinder motion. Therefore, the valve control is essential for the actuator and brings in risks of system failure. Distributed actuation with EHAs proved to simplify the system’s overall complexity and improve flexibility. However, EHAs potential limitations have also gained more and more attention. The EHA configuration, which consists of a set of components, needs more local installation space than a conventional cylinder [3]. Thermal issues emerge as the compact structure integrates heat generating components (motors and pumps) but lacks heat dissipation elements (pipelines) [20].

Li et al. initially proposed a conceptual design of a linear pump with collaborative rectification and discussed the pump composition principles. The internal two pump units were controlled to compress the fluid power and simultaneously rectify for each other. No extra valve control is needed. In addition to the structure simplicity, the advantage of this pump is the flowrate direction control flexibility [21]. Wang et al. used this pump to compose an EHA referred to as a linear-driven EHA (LEHA) and modeled the system by using an energetic macroscopic representation method [22]. This LEHA was kinematically simulated and the capability of its prototype was preliminarily tested [23]. 

**Figure 1:**
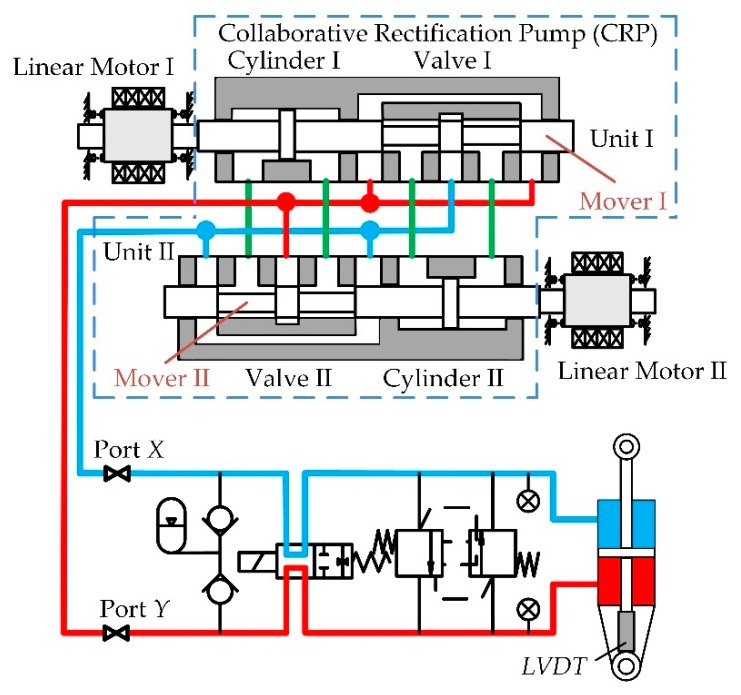
The schematic of the actuator and the collaborative rectification linear pump with two units indicated within the dashed line [23].

The schematic of the actuator is illustrated in Figure 1 [23]. This actuator consists of two linear resonant motors, a linear pump, a hydraulic cylinder, an accumulator, several valves, and sensors. The employed linear resonant motors were designed by Wang et al. with a novel compound Halbach magnet array, and the flux and mover dynamics were analyzed and optimized [24,25,26]. The primary component of the actuator is the linear pump, which incorporates two pump units. Each unit is composed of a miniature cylinder to discharge fluid and a four-way spool valve to rectify the cylinder flow of the other unit. Thus, the pump rectification relies on the collaborative reciprocation of the two movers.

In comparison with conventional rotary driven EHAs, the structure of the proposed EHA is simple and easy to manufacture due to the utilization of the linear pump. The collaborative mechanism ensures the bidirectional driving capability and quick response of active rectification without any additional valve control loop. The objective of this study is to describe the detailed mechanical design, analyze the actuator kinematic and dynamic characteristics, propose a control method based on modulation, and verify the method through experiments of the actuator prototype. The results show that the control method is effective, and the actuator works as expected.

## 2. Design of the Actuator Prototype

### 2.1. Design of the Linear Pump

**Figure 2:**
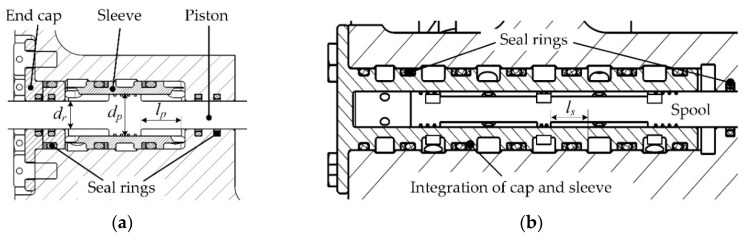
Section views of the linear pump components: (**a**) section view of the cylinder; (**b**) section view of the valve.

The linear pump is the component that logically and physically connects all other components of the actuator (Figure 1). Thus, the linear pump design is primary for the actuator. Technically, the two units can be designed identically and assembled through a manifold. In each unit, the rod of the cylinder and the spool of the valve should be connected. A lever can be used for motion transmission between the rod and the spool. Thus, it is not mandatory that the rod and the spool are coaxial, which facilitates the machining and assembly, and guarantees the sealing effect as well. The section views of the cylinder and valve are shown in Figure 2. According to Figure 2, the effective discharge area of the cylinder is

(1) Ap=14(dp−dr)2π.

**Figure 3:**
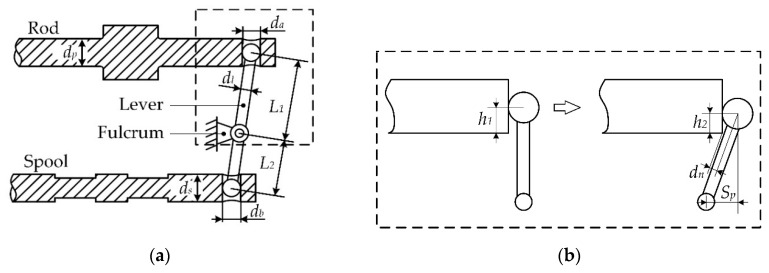
Schematic design of the lever: (**a**) lever between the rod and spool; (**b**) piston moves from neutral to the limit.

The schematic of the lever mechanism and key parameters are shown in Figure 3. 

To avoid mechanical collision between the rod and the lever, and ensure the bulb moves in the groove, the following condition should be satisfied:

(2) dn<12dl and h2>0.

Based on the geometry relationship, the design constraint is

(3) (12da−Sp)L12−Sp2+Sp(L1−h1)L1>dl2,

And the constraint for the spool is deduced similarly.

**Figure 4:**
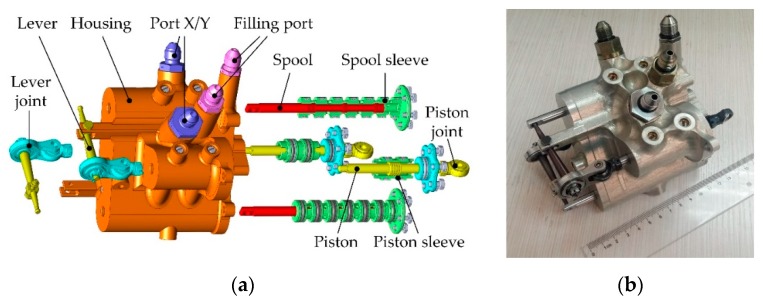
The design of the linear pump: (**a**) exploded view of the pump design; (**b**) prototype of the pump.

The mechanical design of the overall linear pump is shown in Figure 4. Two units of the cylinder and the valve are integrated in one housing, which connects the chambers with internal conduits. The detailed parameters are shown in Table 1.

### 2.2. Integration of the Actuator

**Figure 5:**
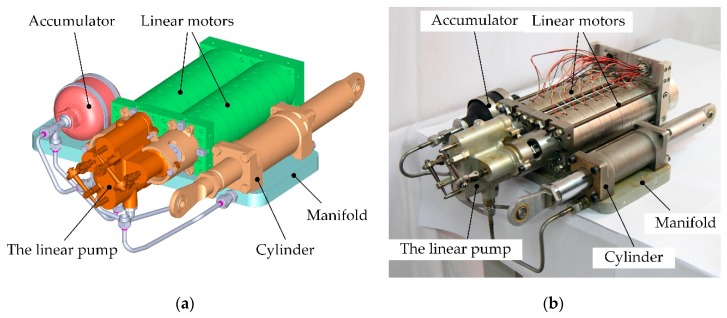
The actuator prototype outline: (**a**) mechanical model of the actuator; (**b**) prototype of the actuator.

The overall actuator design is shown in Figure 5. The linear motor proposed by Jiao and colleagues [24] is hereby employed to drive the linear pump, and two motors are needed according to the pump design. The accumulator is used to prevent the cavitation during the actuator working process. Check valves and relief valves are used to protect the actuator from flow reverse or overload.

The key parameters of this actuator prototype are essential for the following system analysis. They are listed in Table 2.

## 3. Characteristics Analysis of the Actuator

### 3.1. Principle of the Pump Flowrate Variation

Since the driving linear motor mover is a mass-spring system and works at resonant frequency to maximum efficiency, assume that the displacements of the two movers are sinusoid

(4) $$\{\begin{array}{l} x_{1}(t)=S_{m}\sin(2\pi ft)\\ x_{2}(t)=S_{m}\sin(2\pi ft+\varphi ) \end{array},\;\varphi \in \left(-\pi ,\pi \right],$$

where *S_m_* is the stroke of the piston, *f* is the driving reciprocating frequency, and *φ* is the phase difference between the two movers. Thus, the velocities of the movers are

(5) $$\{\begin{array}{l} v_{1}(t)={\dot{x}}_{1}(t)=2\pi fS_{m}\cos(2\pi ft)\\ v_{2}(t)={\dot{x}}_{2}(t)=2\pi fS_{m}\cos(2\pi ft+\varphi ) \end{array},\;\varphi \in \left(-\pi ,\pi \right].$$

We define the displacement of the pump as the flow volume in one motion cycle of the two movers. According to Li and co-workers [21], the defined displacement of the pump is

(6) $$P_{r}=8A_{p}S_{m}\sin(\varphi ).$$

Thus, the flow volume per unit time (i.e., flowrate of the pump) is

(7) $$Q_{r}=8fA_{p}S_{m}\sin(\varphi ).$$

This is the primary principle of the pump output flowrate variation. According to Equation (7), once the pump geometry parameters are fixed, the flowrate is a linear function of the amplitude and frequency of the mover oscillating, and a sine function of the phase difference between the two oscillating movers. Note that in Equation (7), when *f*, *A_p_*, and *S_m_* are defined as positive, the sign of *Q_r_* (i.e., the direction of the flowrate) is only determined by *φ*. This means that when, and only when, *φ* varies across quadrants (between the 1st and 4th quadrant according to the definition in Equation (4)), the pump reverses the flow direction. To maximum the volume efficiency of the pump, the phase difference *φ* should be either π/2 or −π/2.

### 3.2. Resonance Characteristics of the Linear Oscillating Motor

**Figure 6:**
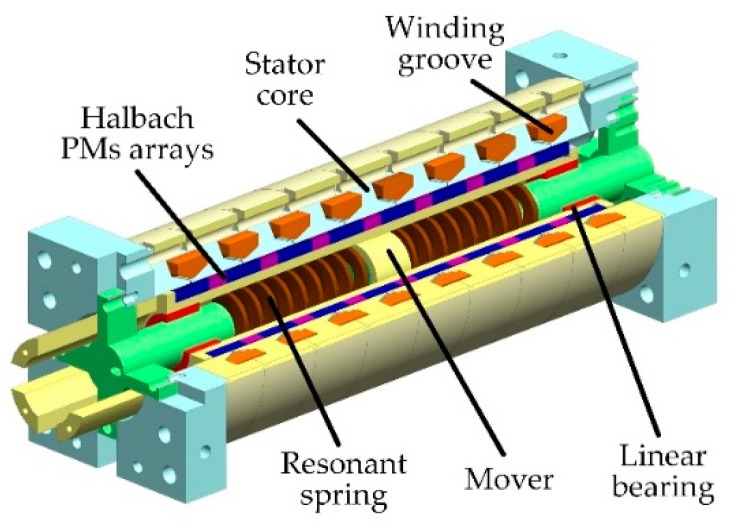
Employed linear oscillating motor [24].

The linear motor internal structure is shown in Figure 6. It is a moving magnet type motor with a Halbach magnet array [24]. The voltage balance equation of the linear motor is

(8) $$U=K_{e}v+R_{m}i+L_{m}\frac{di}{dt},$$

where *U* is the winding voltage, *v* is the mover velocity, *i* is the winding current, and *L_m_* is the winding inductance. The thrust of the linear motor is

(9) $$F_{m}=K_{T}i.$$

The dynamics of the mover consisting of a motor mover, pump piston, and spool are

(10) $$m_{s}\ddot{x}=F_{m}-F_{Lm}-c_{e}\dot{x},$$

where *x* is the displacement of the mover, *F_Lm_* is the load force, and *c_e_* is the damping ratio. To accelerate the mover, the motor thrust should be

(11) $$F_{m}=m_{s}\ddot{x}=4\pi^{2}m_{s}S_{m}f^{2}\sin(2\pi ft).$$

Thus, during one period of the mover oscillation, the motor output power for the acceleration is

(12) $$W_{b}=\int_{T/4}^{T/2}F_{m}vdt+\int_{3T/4}^{T}F_{m}vdt=4\pi^{2}m_{s}S_{m}{}^{2}f^{2}.$$

For a mass-spring system, the resonance frequency is

(13) $$f_{r}=\frac{1}{2\pi }\sqrt{\frac{k_{s}}{m_{s}}},$$

where *k_s_* is the spring constant. Substituting Equation (13) into Equation (12) yields

(14) $$W_{b}=k_{s}S_{m}{}^{2},$$

which represents that by neglecting the damping, the acceleration energy can be transformed into spring mechanical energy, and no extra electromagnetic energy is needed. Since there are two springs mounted symmetrically, each spring constant is $k_{s}/2$.

### 3.3. Overall Dynamics of the Actuator

**Figure 7:**
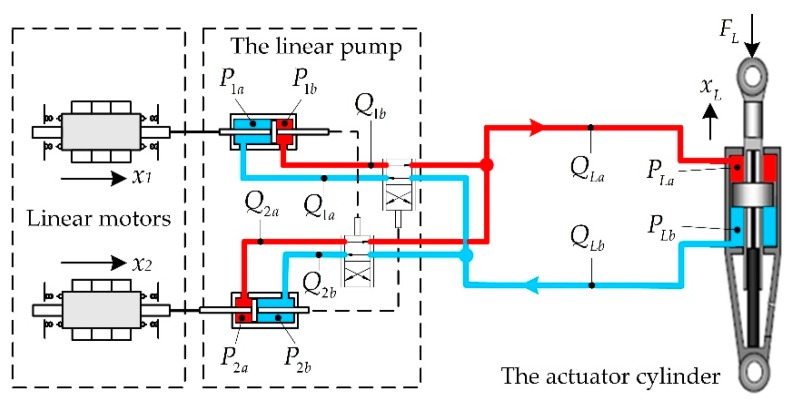
Simplified schematic of the actuator.

A simplified schematic of the actuator is shown in Figure 7. The symbols representing the pressures and flowrates are all labeled. 

The dynamics of the pump cylinders and the actuator cylinder are

(15) $$\{\begin{array}{l} m_{s}{\ddot{x}}_{1}+c_{e}{\dot{x}}_{1}+k_{s}x_{1}=F_{m}+(P_{1a}-P_{1b})A_{p}\\ m_{s}{\ddot{x}}_{2}+c_{e}{\dot{x}}_{2}+k_{s}x_{2}=F_{m}+(P_{2a}-P_{2b})A_{p}\\ m_{L}{\ddot{x}}_{L}+c_{L}{\dot{x}}_{L}=(P_{Lb}-P_{La})A_{L}-F_{L} \end{array},$$

where $c_{L}$ is the damping ratio of the actuator cylinder.

The pressures in the pump cylinder chambers are

(16) $$\{\begin{array}{l} {\dot{P}}_{na}=\frac{\beta }{V_{na}}(A_{p}{\dot{x}}_{n}-Q_{na})\\ {\dot{P}}_{nb}=-\frac{\beta }{V_{nb}}(A_{p}{\dot{x}}_{n}-Q_{nb}) \end{array},n=1,2,$$

where *β* is the fluid elastic modulus, and *V_na_* and *V_nb_* are the volume of the pump cylinder chambers. Similarly, the pressures in the actuator cylinder chambers are

(17) $$\{\begin{array}{l} {\dot{P}}_{La}=\frac{\beta }{V_{La}}(A_{L}{\dot{x}}_{L}-Q_{La})\\ {\dot{P}}_{Lb}=-\frac{\beta }{V_{Lb}}(A_{L}{\dot{x}}_{L}-Q_{Lb}) \end{array},$$

where *V_La_* and *V_Lb_* are the volumes of the actuator cylinder chambers.

## 4. Control of the Actuator

**Figure 8:**
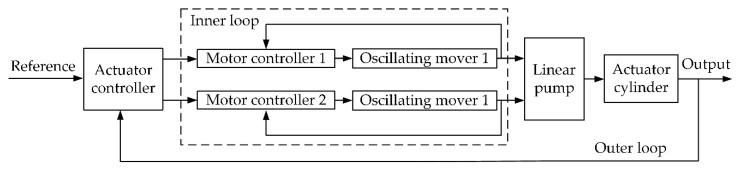
The inner loop and outer loop of the actuator control.

According to the above analysis, the control of the actuator can be divided into two loops as shown in Figure 8. The inner loop controls the linear motors to oscillate at the resonant frequency. The outer loop controls the actuator cylinder to follow the actuator position reference from the system input.

### 4.1. The Linear Motor Mover Control of the Inner Loop

**Figure 9:**
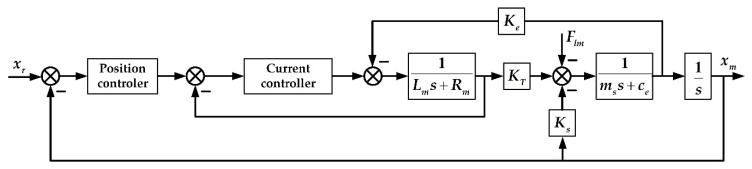
Cascaded control of the linear oscillating motor.

According to Section 3.2, the linear oscillating motor control block diagram with a cascaded control architecture is shown in Figure 9.

In order to ensure the mover oscillates at the resonant frequency, a feedforward controller is employed here to compensate the disturbance of the mechanical load and viscosity. Since the mover is a mass-spring system and fluid compression is equivalent to a spring, the feedforward controller is designed as

(18) $$u_{a}(t)=P_{1}{\dot{x}}_{r}(t)+P_{2}x_{r}(t),$$

where $P_{1}$ is the damping coefficient and $P_{2}$ is the elastic coefficient. The position controller and current controller are both proportional–integral–derivative (PID) controllers due to engineering practicability.

### 4.2. The Actuator Position Control of the Outer Loop

**Figure 10:**
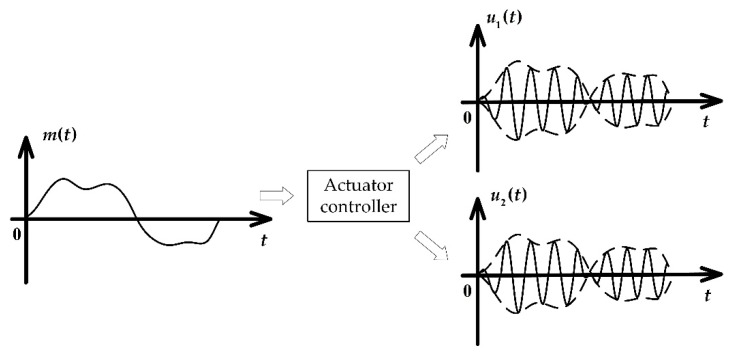
The actuator controller modulates the input reference onto the sine signal of the linear motor resonant frequency.

According to Section 3.1, the pump flowrate is determined by the collaborative oscillation of the two movers. However, the resonance requires a particular working frequency (i.e., the variable *f* should not vary once the mover mass and spring stiffness are fixed). Hence, the pump flow control mainly relies on the amplitude regulation of the mover oscillation. This means that the actuator controller needs to transform the input into two channels of oscillating signal and output them to the two linear motors respectively. Therefore, the actuator controller contains the function of amplitude modulation and phase modulation as shown in Figure 10.

With reference to a study by Ziemer and Tranter [27], a double-sideband suppressed-carrier (DSB-SC) is the most appropriate amplitude modulation (AM) for the actuator control. No offset component is included in the modulated signal and the sine waveform is complete, for no spectrum is filtered. 

The flow direction is controlled by the phase difference *φ*. It is not necessary to vary both phases of the two channels of the oscillating signal. Hereby only the phase of unit Ⅱ oscillating signal is modulated, which is a special case of phase modulation (PM) as the phase is modulated to be either π/2 or −π/2.

**Figure 11:**
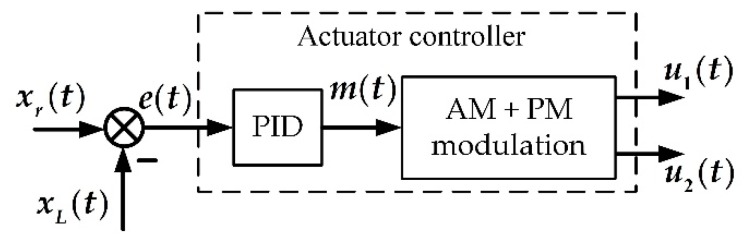
The actuator controller is composed of a series of a PID controllers and an amplitude modulation (AM) + phase modulation (PM) modulator.

Therefore, the actuator controller is designed as a series of PID controllers followed by a modulation block as shown in Figure 11.

According to the algorithm of PID and DSB-SC, the controller is described as

(19) $$\{\begin{array}{l} u_{1}(t)=(K_{p}e(t)+K_{i}\int_{0}^{t}e(t^{\prime })dt^{\prime }+K_{d}\frac{de(t)}{dt})\cdot \sin(2\pi ft)\\ u_{2}(t)=(K_{p}e(t)+K_{i}\int_{0}^{t}e(t^{\prime })dt^{\prime }+K_{d}\frac{de(t)}{dt})\cdot \sin(2\pi ft+\mathrm{sgn}(e(t))\cdot \frac{\pi }{2}) \end{array},$$

where

(20) $$\mathrm{sgn}(e(t))=\{\begin{array}{ll} 1 & e(t)>0\\ 0 & e(t)=0\\ -1 & e(t)<0 \end{array}.$$

## 5. Experiments and Verification

**Figure 12:**
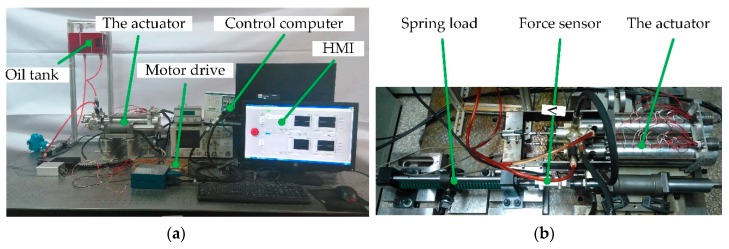
The actuator test rig: (**a**) the overall test rig with the actuator control system; (**b**) the actuator output force testing configuration.

The test rig is established as shown in Figure 12. The data acquisition and control system are developed upon a LabVIEW RT platform running on PXI-1036 computer produced by the National Instruments Corporation. The linear motor position is measured by a linear variable differential transformer with a range of 68 mm, while the actuator cylinder position is measured by a similar model with a 650 mm range. The linear motor drive is a pulse width modulation (PWM) brush-type motor servo drive, and the model is AMC 50A20I.

In order to verify the actuator principle and control method, three experiments were carried out:

1. Linear motor control experiment. The aim was to verify the linear motor resonant oscillating feasibility, and the effectiveness of the proposed PID + feedforward control method.
2. The actuator tracking performance experiment. The aim was to verify the pump and actuator control feasibility and the tracking performance of the actuator control.
3. The actuator spring load experiment. The aim was to verify the actuator performance with a spring load disturbance and test the actuator force capability with dynamic loads.

### 5.1. Linear Motor Control Experiment

The control of the linear motor mover position is a fundamental of the actuator control. A controllable and stable resonance guarantees the displacement control of the pump. This part of the linear motor control experiment separates into two stages: Firstly, the motor oscillation is tested by using the cascaded closed-loop control architecture without the feedforward; secondly, the feedforward control is added to verify its performance.

The linear motor of the actuator prototype is designed to oscillate at a frequency of 30 Hz. According to Section 3.2, the stiffness of each internal spring is

(21) $$k_{s1}=k_{s2}=\frac{k_{s}}{2}=\frac{4\pi^{2}f_{r}{}^{2}m_{s}}{2}=20430\;(N/m).$$

**Figure 13:**
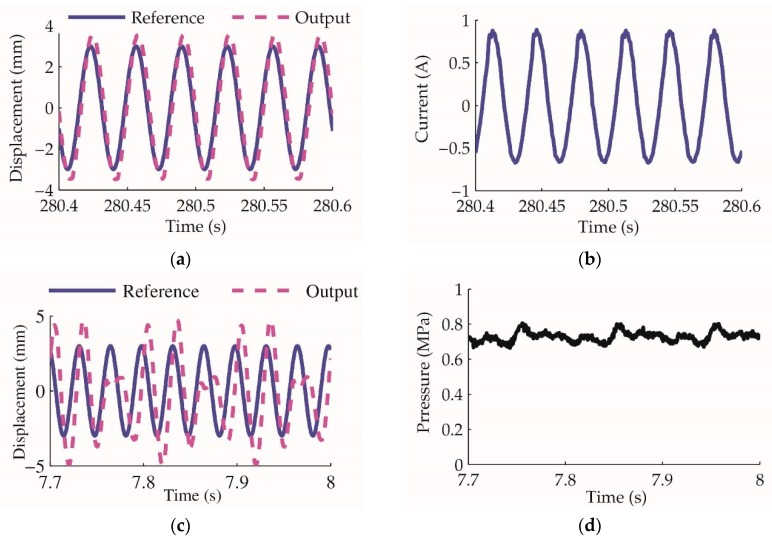
Linear motor oscillation closed-loop control experiment: (**a**) displacement reference and output without load; (**b**) current of the motor winding without load; (**c**) displacement reference and output with load; (**d**) the pressure acted on the motor mover is approximately 0.7 MPa, and for the effective area of the mover piston it is 48.1 mm^2^ according to Table 2, the equivalent force is 34 N.

The experiment result of closed-loop control without feedforward is shown in Figure 13. The reference command is a sine waveform of 3 mm stroke and 30 Hz frequency. It is demonstrated that the linear motor oscillates smoothly under the closed-loop control without any load. The current peak value is less than 1 A. The energy consumed by the motor is mainly used to overcome the mechanical viscosity. When an external load acts on the motor mover, the oscillation is disturbed seriously even when the equivalent force is only approximately 34 N. This means the linear motor oscillation is weak in resisting external disturbance.

**Figure 14:**
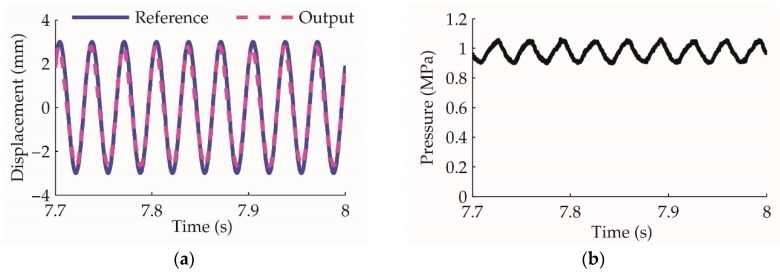
After applying the feedforward control on the linear motor, the mover oscillation experiment: (**a**) displacement reference and output with load; (**b**) the pressure acts on the motor mover, which is equivalent 48 N.

After applying the feedforward control proposed in Section 4.1, the experiment results are shown in Figure 14. It demonstrates that even as the load increases, the motor mover oscillates ideally under the improved control method. The feedforward is effective for the linear motor oscillation control and can be used as the inner loop of the overall actuator control.

### 5.2. The Actuator Tracking Performance Experiment

**Figure 15:**
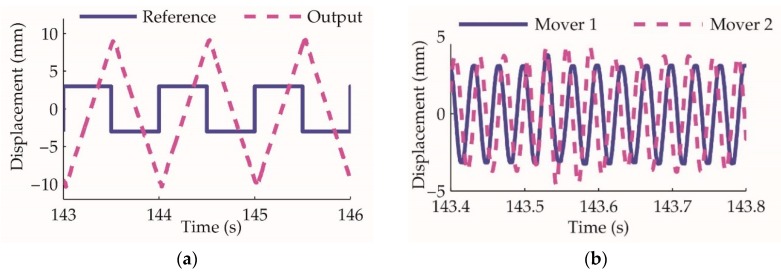
The actuator open-loop control experiment: (**a**) the actuator cylinder position reference and output; (**b**) displacement of the two movers, at approximately 143.55 s, mover Ⅱ phase varies from π/2 to −π/2, and the actuator cylinder is driven reversely.

Based on the linear oscillating motor control, the actuator working principle is verified through the collaboration of the two-unit movers. When the actuator is under open-loop control and the reference signal is a square wave, the amplitude of the modulated control signals of the two motors is constant, and the phase varies between π/2 and −π/2. This experiment result is shown in Figure 15. The actuator piston moves at a constant speed for the constant motor oscillating amplitude. The phase difference between the two movers varying from π/2 to −π/2 drives the actuator reversely, which verify the collaborative rectification principles.

As shown in the results, the actuator cylinder moves at 38.7 mm/s, which means the pump output flowrate is 2.32 L/min. According to the design parameters, the theoretical flowrate is 2.4 L/min, demonstrating that the implemented prototype output flow is close to the theoretical design.

**Figure 16:**
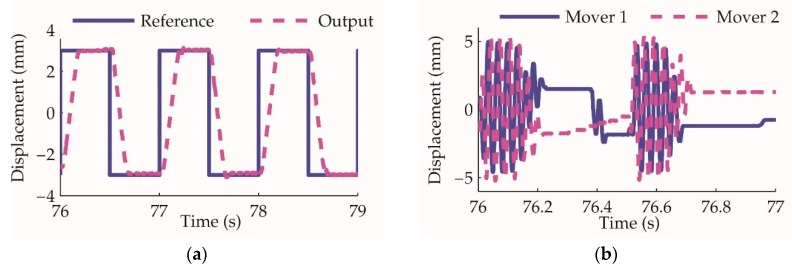
The actuator closed-loop control experiment: (**a**) the actuator cylinder position reference and output; (**b**) displacement of the two movers, the amplitude modulation and phase modulation is demonstrated.

By employing the controller designed in Section 4.2, the actuator closed-loop control experiment results are shown in Figure 16. At the initial stage of the step response, the movers oscillate at the maximum range of ±5 mm and provide the maximum flowrate to drive the cylinder to track the reference as fast as possible. With the output approaching the reference, the amplitude decreases to 0, and the actuator cylinder is locked at the desired position.

**Figure 17:**
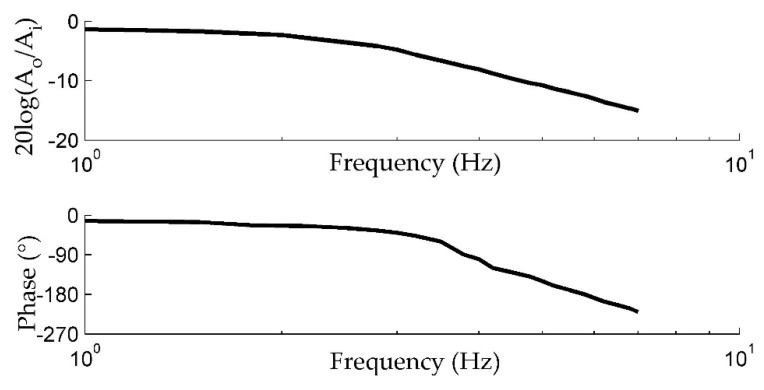
The frequency response of the closed-loop actuator.

In order to obtain the frequency response of the actuator, a sweep frequency analysis is conducted. The bode plot is shown in Figure 17. 

### 5.3. The Actuator Spring Load Experiment

**Figure 18:**
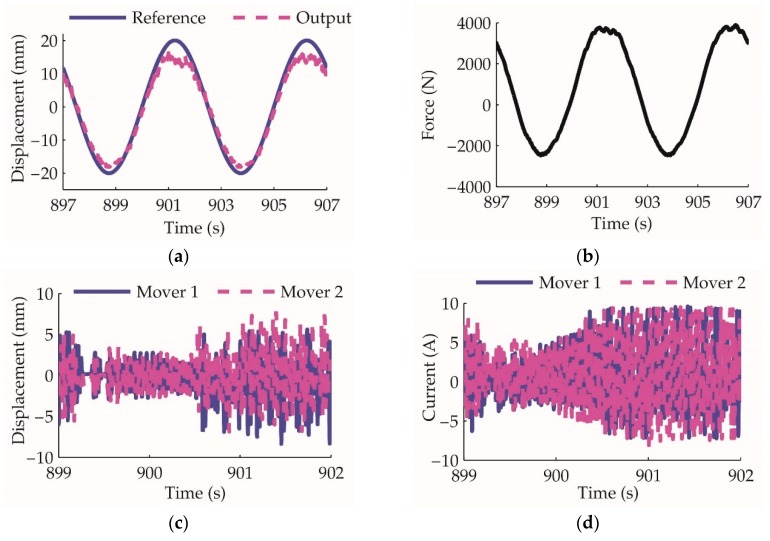
The actuator spring load experiment: (**a**) displacement reference and output of the actuator; (**b**) output force of the actuator; (**c**) displacement reference and output of the two movers; (**d**) the current of the two linear motors.

In many application scenarios of actuation, the load can be emulated by a spring. Hereby, the load experiment uses a spring as the external load of the actuator. The mechanical connection is shown in Figure 12b. The control command is a sine waveform with ±20 mm amplitude and 0.2 Hz frequency, and the results are shown in Figure 18.

From Figure 18d, when the dynamic load reaches approximately 4000 N, the current of the linear motor approaches 8 A, which is the designed maximum current capacity of the motor. According to the actuator design parameters, the theoretical maximum output force is nearly

(22) $$\frac{F_{m}A_{L}}{A_{p}}=\frac{K_{T}iA_{L}}{A_{p}}\approx 4400\;(N),$$

which means the prototype output force capability is close to the design.

## 6. Discussion

In order to provide a simplified and low-cost terminal solution for a distributed actuation system, this paper proposes an electro-hydrostatic actuator based on the linear drive principle. A novel collaborative rectification mechanism is designed to actively rectify the involved linear pump efficiently. A compact prototype is designed and the detailed structure is revealed. The actuator working principle based on the structure is analyzed and a dedicated control architecture is proposed. The control method guarantees the actuator is working as designed, which is verified by experiments on different levels of the prototype system. Based on the verified structure and control method, a more comprehensive and sophisticated design will be carried out in the future.

## Figures and Tables

**Table 1:** Mechanical parameters of the pump.

Items	Symbol	Value
Diameter of rod	*dr*	7 (mm)
Diameter of piston	*dp*	10.5 (mm)
Diameter of spool	*ds*	7 (mm)
Length of lever	*L*_1_+*L*_2_	51.7 (mm)
Ratio of lever	*L*_1_/*L*_2_	1.7
Maximum stroke of piston	*lp*	10 (mm)
Maximum stroke of spool	*ls*	8.7 (mm)

**Table 2:** Key design parameters of the actuator.

Items	Symbol	Value
Force constant of the motor	*K_T_*	27.5 (N/A)
Back electromotive force (EMF) constant of the motor	*K_e_*	27.5 (V∙s/m)
Resistance of the motor winding	*R_m_*	2.2 (Ω)
Inductance of the motor winding	*L_m_*	12 (mH)
Equivalent mass of the mover (motor and the pump)	*m_s_*	1.15 (kg)
Stroke of the motor	*S_m_*	±5 (mm)
Effective area of the pump piston	*A_p_*	48.1 (mm^2^)
Effective area of the actuator cylinder	*A_L_*	954 (mm^2^)
Mass of the actuator cylinder piston	*m_L_*	1.1 (kg)
Stroke of the actuator cylinder	*S_L_*	±37 (mm)

## Nomenclature

**Symbol**	**Items**
*A_p_*	Effective area of the pump piston
*d_r_*	Diameter of the pump rod
*d_p_*	Diameter of the pump piston
*d_s_*	Diameter of the pump spool
*L*_1_	Lever length of the pump cylinder piston
*L*_2_	Lever length of the pump valve spool
*l_p_*	Maximum stroke of piston
*l_s_*	Maximum stroke of spool
*d_n_*	Minimum distance between the pump piston rod and the lever
*d_l_*	Diameter of the pump lever
*h*_1_	Distance between the pump piston rod excircle and lever bulb center at the neutral position
*h*_2_	Distance between the pump piston rod excircle and lever bulb center at the limit position
*S_p_*	Horizontal distance between two bulbs of the lever
*K_T_*	Force constant of the motor
*K_e_*	Back EMF constant of the motor
*R_m_*	Resistance of the motor winding
*L_m_*	Inductance of the motor winding
*m_s_*	Equivalent mass of the mover (motor and the pump)
*S_m_*	Stroke of the motor
*A_p_*	Effective area of the pump piston
*A_L_*	Effective aera of the actuator cylinder
*m_L_*	Mass of the actuator cylinder piston
*S_L_*	Stroke of the actuator cylinder
*S_m_*	Stroke of the pump piston
*f*	Frequency of the pump piston reciprocation
*φ*	Phase difference between the two pump movers
*P_r_*	Flowrate of the pump per period
*Q_r_*	Flowrate of the pump per unit time
*U*	Voltage of the motor winding
*v*	Velocity of the motor mover
*i*	Current of the motor winding
*F_m_*	Thrust of the motor
*x*	Displacement of the motor mover
*F_Lm_*	Load force of the motor mover
*c_e_*	Damping ratio of the motor mover
*W_b_*	Motor output power per period
*k_s_*	Spring constant
*c_L_*	Damping ratio of the actuator cylinder
*F_L_*	Load force of the actuator cylinder
*A_L_*	Effective area of the actuator cylinder
*β*	Elastic modulus of fluid, are the
*V_na_*, *V_nb_*	Volumes of the pump cylinder chambers
*Q_na_*, *Q_nb_*	Flowrate of the pump cylinder chambers
*P_na_*, *P_nb_*	Pressure of the pump cylinder chambers
*V_La_*, *V_Lb_*	Volumes of the actuator cylinder chambers
*Q_La_*, *Q_Lb_*	Flowrate of the actuator cylinder chambers
*P_La_*, *P_Lb_*	Flowrate of the actuator cylinder chambers
*P*_1_	Damping coefficient of the feedforward
*P*_2_	Elastic coefficient of the feedforward
